# Edible plants as significant sources of *Blastocystis* spp. infections: A systematic review and meta-analysis

**DOI:** 10.1016/j.fawpar.2025.e00254

**Published:** 2025-03-02

**Authors:** Abdollah Didban, Farajolah Maleki, Laya Shamsi, Ali Asghari, Behzad Bijani, Amin Karampour

**Affiliations:** a Medical Microbiology Research Center, Qazvin University of Medical Sciences, Qazvin, Iran; b Zoonotic Diseases Research Center, Ilam University of Medical Sciences, Ilam, Iran; c Clinical Research Development Unit, Shahid Mostafa Khomeini Hospital, Ilam University of Medical Sciences, Ilam, Iran; d Department of Pathobiology, Faculty of Veterinary Medicine, Urmia University, Urmia, Iran.

**Keywords:** *Blastocystis* spp., Prevalence, Subtypes, Foods, Plants, Vegetables

## Abstract

This systematic review and meta-analysis were conducted to comprehensively overview the global epidemiology and subtypes (STs) distribution of *Blastocystis* spp. in edible plants. A comprehensive search of various electronic databases (PubMed, Scopus, Google Scholar, and Web of Science) until May 19, 2024, found 27 studies/41 datasets meeting inclusion criteria, covering 8794 edible plants from 15 countries globally. Examined edible plants were composed of fruits (six datasets, 1198 samples), non-leafy green vegetables (10 datasets, 1158 samples), leafy green vegetables (18 datasets, 4245 samples), and uncategorized plants (seven datasets, 2193). This study revealed that 9.4 % (95 % CI: 6.6–13.4 %) of global edible plants harbored *Blastocystis* spp. Fruits had the highest *Blastocystis* spp. contamination rate among edible plants at 12.5 % (95 % CI: 5.4–26.6 %), followed by uncategorized plants at 10.2 % (95 % CI: 4.5–21.5 %), leafy green vegetables at 9.3 % (95 % CI: 6.1–13.9 %), and non-leafy green vegetables at 5.6 % (95 % CI: 2.5–12.1 %). Sensitivity analysis evaluated weighted prevalence changes after excluding specific studies. Subgroup analysis was conducted considering publication years, countries, continents, WHO regions, sample sizes, and diagnostic methods. Interestingly, zoonotic STs (ST1 and ST3) of *Blastocystis* spp. have been found in edible plants. These results highlight the potential risk of *Blastocystis* spp. transmission through consuming contaminated edible plants, emphasizing the importance of implementing adequate food safety measures to decrease the prevalence of this parasite in the food chain.

## Highlights

• This study revealed that 9.4 % (95 % CI: 6.6–13.4 %) of global edible plants may harbour *Blastocystis* spp.
• Fruits had the highest contamination rate of *Blastocystis* spp. among edible plants at 12.5 % (95 % CI: 5.4–26.6 %).
• Zoonotic *Blastocystis* spp. subtypes ST1 and ST3 were reported in edible plants.
• This study highlights the potential risk of *Blastocystis* spp. transmission via consumption of contaminated edible plants.

## 1 Introduction

*Blastocystis* is an enteric protistan parasite that infects people and various animals, raising worries about its potential spread through contaminated food, particularly edible plants including fruits, leafy green vegetables, and non-leafy green vegetables (Licea et al., 2003; Rauff-Adedotun et al., 2021). This enteric parasite is a cause of waterborne and foodborne infections (Ghafari-Cherati et al., 2024; Mahdavi et al., 2024).

The *SSU-rRNA* gene polymorphism has identified 44 genetically different variants or subtypes (STs) of *Blastocystis* spp. Sixteen have been found in humans and animals (ST1-ST10, ST12, ST14, ST16, ST23, ST35, and ST41), with ST1–ST4 making up >90 % of human isolates. Most ST1–ST4 infections are typically transmitted between humans, whereas other STs are more common in specific host groups like mammals or birds, being spread through human–animal interactions (Koehler et al., 2024; Santin et al., 2024).

Previous reports indicate that infection with pathogenic *Blastocystis* spp. ST7 is linked to reduced bacterial diversity and altered gut microbiome profiles in patients with diarrhea (Deng et al., 2022). Furthermore, colonization with ST1 and ST4, two prevalent STs in humans, can positively influence host health by modulating gut microbiota and adaptive immune responses (Deng et al., 2023a, Deng et al., 2023b).

Fruits and vegetables are key in transmitting parasitic diseases like *Blastocystis* spp. due to their ability to carry and spread infectious agents. They can get parasites from water, soil, or animal waste during growing, picking, or processing. Eating raw, undercooked, or unwashed contaminated produce can pass parasitic infections to people (Badri et al., 2022; Li et al., 2020). Several studies have reported the presence of *Blastocystis* spp. in edible plants including leafy green vegetables, non-leafy green vegetables, and fruits (Table 1, Table 2). In this paper, we conducted a statistical analysis and systematic review of the epidemiology and subtype distribution of *Blastocystis* spp. in edible plants from a global standpoint.

**Table 1:** The main information from 27 studies/41 datasets included in the present systematic review on the prevalence and STs distribution of *Blastocystis* spp. in edible plants.

Author, year	Examined plants	Most infected plant	Time tested	Country	Total no.	Infected no.	Prevalence (%)	Method	STs ^g^ (no./%)
Soares and Cantos, 2006	LGVs ^a^	UC ^c^	2003–2004	Brazil	750	146	19.5	Mic ^e^	–
Al-Megrin, 2010	LGVs	UC	UC	Saudi Arabia	470	13	2.8	Mic	–
Cazorla Perfetti et al., 2013	LGVs, fruits	UC	2009	Venezuela	45	10	22.2	Mic	–
Rahimi Esboei et al., 2017	LGVs, NLGVs ^b^	UC	2012	Iran	150	12	8	Mic	–
Caradonna et al., 2017	LGVs, NLGVs	UC	2015–2016	Italy	648	3	0.5	Mol ^f^	–
Etewa et al., 2017	LGVs, NLGVs, fruits	Straw berry	2016	Egypt	420	16	3.8	Mic	–
Taherimoghaddam et al., 2018	LGVs, NLGVs	Parsley	2017–2018	Iran	380	3	0.8	Mic	–
Isazadeh et al., 2020	LGVs, NLGVs	Basil	2017–2018	Iran	240	10	4.2	Mic	–
Rodrigues et al., 2020	LGVs	Lettuce	2018	Brazil	200	67	33.5	Mic	–
Heidari et al., 2020	LGVs, NLGVs	Cress	2017	Iran	120	12	10	Mic	–
Al Nahhas and Aboualchamat, 2020	LGVs, NLGVs	Lettuce	2019	Syria	128	13	10.1	Mol	–
Aydoğdu et al., 2021	LGVs, NLGVs, fruits	UC	UC	Turkey	80	12	15	Mol	ST1 (12/100)
Rahimi et al., 2021	LGVs, NLGVs	Spinach, Dill ^d^	2017	Iran	196	24	12.2	Mic	–
Abdel-Hakeem et al., 2021	LGVs	Arugula	2018–2019	Egypt	270	21	7.8	Mic	–
Morales-Figueroa et al., 2021	NLGVs, fruits	Asparagus, Grape	2019–2020	Mexico	400	37	9.2	Mic	–
Hussein et al., 2021	LGVs, NLGVs	Spinach, Dill, Red Radish	2018–2019	Saudi Arabia	400	11	2.7	Mic	–
Hoseinifard et al., 2022	LGVs, NLGVs	Basil	2021	Iran	160	4	2.5	Mic	–
Abd Rabou et al., 2022	LGVs, fruits	UC	2020–2021	Egypt	1000	42	4.2	Mic	–
Jinatham et al., 2022	NLGVs, fruits	UC	UC	Thailand	20	9	45	Mol	ST3 (7/77.8), UN ^h^ (2/22.2)
El Safadi et al., 2023	LGVs	UC	2020–20,201	Lebanon	300	26	8.7	Mic	–
Falcone et al., 2023	LGVs	UC	2017–2020	Argentina	261	63	24.1	Mic	–
Moreno-Mesonero et al., 2023	LGVs, fruits	Lettuce	2020	Spain	110	2	1.8	Mol	–
Bilgiç et al., 2023	LGVs, fruits	Dill	UC	Turkey	80	4	5	Mic	–
Barua et al., 2023	Fruits	Tamarind water	2021–2022	Bangladesh	200	66	33	Mic	–
Altwaim et al., 2023	LGVs, NLGVs	Lettuce	2020–2021	Saudi Arabia	250	49	19.6	Mic	–
El-Sayed et al., 2023	LGVs	Dill, Coriander	2020	Egypt	100	34	34	Mic	–
González-Ramírez et al., 2023	LGVs, NLGVs, fruits	Straw berry	UC	Ecuador	1416	475	33.5	Mic	–

^a^ LGVs: Leafy green vegetables (Plants with leaves), ^b^ NLGVs: Non-leafy green vegetables (Plants without leaves), ^c^ UC: Unclear, ^d^ More than one plant means an equal prevalence rate, ^e^ Mic: Microscopic detection, ^f^ Mol: Molecular detection, ^g^ STs: Subtypes, ^h^ UN: Unidentified.

**Table 2:** Global distribution and prevalence of *Blastocystis* spp. in different types of edible plants.

Author, year	Fruits			NLGVs ^a^			LGVs ^b^			Uncategorized plants ^c^			Total		
	Total no.	Infected no.	%	Total no.	Infected no.	%	Total no.	Infected no.	%	Total no.	Infected no.	%	Total no.	Infected no.	%
Soares and Cantos, 2006	–	–	–	–	–	–	750	146	19.5	–	–	–	750	146	19.5
Al-Megrin, 2010	–	–	–	–	–	–	470	13	2.8	–	–	–	470	13	2.8
Cazorla Perfetti et al., 2013	–	–	–	–	–	–	–	–	–	45	10	22.2	45	10	22.2
Rahimi Esboei et al., 2017	–	–	–	–	–	–	–	–	–	150	12	8	150	12	8
Caradonna et al., 2017	–	–	–	–	–	–	–	–	–	648	3	0.5	648	3	0.5
Etewa et al., 2017	140	7	5	70	3	4.3	210	6	2.8	–	–	–	420	16	3.8
Taherimoghaddam et al., 2018	–	–	–	114	1	0.9	266	2	0.7	–	–	–	380	3	0.8
Isazadeh et al., 2020	–	–	–	72	3	4.2	168	7	4.2	–	–	–	240	10	4.2
Rodrigues et al., 2020	–	–	–	–	–	–	200	67	33.5	–	–	–	200	67	33.5
Heidari et al., 2020	–	–	–	60	3	5	60	9	15	–	–	–	120	12	10
Al Nahhas and Aboualchamat, 2020	–	–	–	16	1	6.2	112	12	10	–	–	–	128	13	10.1
Aydoğdu et al., 2021	–	–	–	–	–	–	–	–	–	80	12	15	80	12	15
Rahimi et al., 2021	–	–	–	30	2	6.7	166	22	13.2	–	–	–	196	24	12.2
Abdel-Hakeem et al., 2021	–	–	–	–	–	–	270	21	7.8	–	–	–	270	21	7.8
Morales-Figueroa et al., 2021	300	22	7.3	100	15	15	–	–	–	–	–	–	400	37	9.2
Hussein et al., 2021	–	–	–	112	4	3.6	288	7	2.4	–	–	–	400	11	2.7
Hoseinifard et al., 2022	–	–	–	96	1	1	64	3	4.7	–	–	–	160	4	2.5
Abd Rabou et al., 2022	–	–	–	–	–	–	–	–	–	1000	42	4.2	1000	42	4.2
Jinatham et al., 2022	–	–	–	–	–	–	–	–	–	20	9	45	20	9	45
El Safadi et al., 2023	–	–	–	–	–	–	300	26	8.7	–	–	–	300	26	8.7
Falcone et al., 2023	–	–	–	–	–	–	261	63	24.1	–	–	–	261	63	24.1
Moreno-Mesonero et al., 2023	22	0	0	–	–	–	88	2	2.3	–	–	–	110	2	1.8
Bilgiç et al., 2023	20	0	0	–	–	–	60	4	6.7	–	–	–	80	4	5
Barua et al., 2023	200	66	33	–	–	–	–	–	–	–	–	–	200	66	33
Altwaim et al., 2023	–	–	–	–	–	–	–	–	–	250	49	19.6	250	49	19.6
El-Sayed et al., 2023	–	–	–	–	–	–	100	34	34	–	–	–	100	34	34
González-Ramírez et al., 2023	516	193	37.4	488	134	27.5	412	148	35.9	–	–	–	1416	475	33.5

^a^ NLGVs: Non-leafy green vegetables (plants without leaves), ^b^ LGVs: Leafy green vegetables (plants with leaves), ^c^ Contaminated vegetable foods that were not categorized as fruits, non-leafy green vegetables, and/or leafy green vegetables.

## 2 Methods

### 2.1 Ethics approval

The present study was approved by the Ethics Committee of Qazvin University of Medical Sciences, Qazvin, Iran (approval no. IR.QUMS.REC.1403.175).

### 2.2 Study design

The current study was a worldwide systematic review and meta-analysis of *Blastocystis* spp. prevalence and its subtype distribution in edible plants. This study was conducted in 2024. The reporting approach for this study follows the PRISMA (Preferred Reporting Items for Systematic Reviews and Meta-Analysis) checklist (Moher et al., 2015).

### 2.3 Search strategy

The researchers in the study examined four international databases: Medline/PubMed, Scopus, and the Web of Knowledge, for articles published up to May 19, 2024. Additionally, Google Scholar was searched for grey literature. The search was conducted using Medical Subject Heading (MeSH) terms alone or in combination: (“Intestinal Parasites” OR “Parasitic Infections” OR “*Blastocystis* spp.”) AND (“Prevalence” OR “Epidemiology” OR “Frequency” OR “Occurrence”) AND (“Subtype” OR “Subtyping”) AND (“Foods” OR “Plants” OR “Vegetables” OR “Fruits”). To include more pertinent papers, extra keywords were utilized, and the references of relevant papers were examined. The data collected was input into EndNote X7 software, and duplicate articles were automatically removed. It is important to mention that two researchers independently reviewed the articles.

### 2.4 Inclusion and exclusion criteria

Studies from all languages, regions, and time periods that reported *Blastocystis* spp. prevalence in edible plants using microscopy, molecular, and serological methods were assessed in this global review. Excluded were animal, human, and non-plant studies, case reports, reviews, and studies lacking total sample size and/or *Blastocystis* spp. prevalence rate.

### 2.5 Quality evaluation

Papers underwent qualitative assessment for inclusion/exclusion using the Joanna Briggs Institute (JBI) Critical Appraisal Checklist for Studies Reporting Prevalence Data (Porritt et al., 2014). Studies with scores of ≤4–6 and ≥ 7 points were categorized as moderate-quality and high-quality, respectively. Articles scoring ≤3 were excluded. Two researchers extracted essential data from the selected papers, which were verified by other researchers. Extracted information comprised the last name of the primary author, type of plant, assessment score for quality, year of publication, year of implementation, continent, country, World Health Organization (WHO) classification, total sample size, and the number of samples infected.

### 2.6 Meta-analysis

All statistical analyses were conducted using the Comprehensive Meta-Analysis (CMA) v3 software. *P*-values <0.05 were considered statistically significant. The random-effects model was used to assess the prevalence of *Blastocystis* spp. in edible plants by estimating pooled prevalence and 95 % CIs. Subgroup analysis was performed to evaluate the weighted prevalence of infection in edible plants based on plant types, WHO regions, countries, publication years, continents, sample size, and diagnostic methods. A forest plot diagram was generated to illustrate the pooled prevalence with 95 % CIs. The funnel plot was employed to examine publication bias in the analysis. Heterogeneity among studies was assessed using the *I*^*2*^ index, where values below 25 %, 25–50 %, and over 50 % were classified as low, moderate, and high heterogeneity, respectively. Additionally, sensitivity analysis was carried out to assess variations in the final weighted prevalence of *Blastocystis* spp. after excluding individual studies.

## 3 Results

### 3.1 Paper selection

Expert researchers rigorously searched four international databases and obtained a total of 7925 initial records. Following deduplication and a thorough review of the remaining papers (4682 records), 31 articles were ultimately selected. Furthermore, a quality assessment using JBI criteria resulted in the exclusion of four additional studies. Ultimately, 27 highly pertinent papers (41 datasets) satisfied the inclusion criteria for this study (Supplementary Fig. 1).

### 3.2 Qualitative and quantitative features of the papers included

This study covered 27 articles [41 datasets (six on fruits, 10 on non-leafy green vegetables, 18 on leafy green vegetables, and seven on uncategorized plants) from 2006 to 2024. The 8794 edible plants consisted of 1198 fruits, 1158 non-leafy green vegetables, 4245 leafy green vegetables, and 2193 uncategorized plants. Geographically, Iran and Egypt lead with six and four studies, followed by Saudi Arabia (three), Brazil and Turkey (two each), and Argentina, Bangladesh, Ecuador, Italy, Lebanon, Mexico, Spain, Syria, Thailand, and Venezuela (one each). The sample sizes varied from 20 to 1416 edible plants. Among the 27 studies, only two papers provided detailed information on the subtype distribution of *Blastocystis* spp. in edible plants. Microscopy was the most commonly used diagnostic method in 22 studies, while molecular techniques were used in five studies (Table 1). The quality assessment using the JBI checklist revealed that 16 papers were of high quality (>6 points), and the remaining 11 articles were of moderate quality (4–6 points) (Supplementary Table 1).

### 3.3 Global epidemiology of *Blastocystis* spp. in edible plants

This study revealed that 9.4 % (95 % CI: 6.6–13.4 %) of global edible plants harbored *Blastocystis* spp. (Supplementary Fig. 2). Statistical analysis revealed a considerable degree of heterogeneity in the current systematic review and meta-analysis (Q = 766.5, *I*^*2*^ = 96.6 %, *P* < 0.001).

### 3.4 Weighted prevalence of *Blastocystis* spp. based on plant types

Among edible plants, fruits exhibited the highest *Blastocystis* spp. contamination rate at 12.5 % (95 % CI: 5.4–26.6 %), surpassing uncategorized plants at 10.2 % (95 % CI: 4.5–21.5 %), leafy green vegetables at 9.3 % (95 % CI: 6.1–13.9 %), and non-leafy green vegetables at 5.6 % (95 % CI: 2.5–12.1 %) (Table 2 and Supplementary Fig. 3).

### 3.5 Pooled prevalence of *Blastocystis* spp. in edible plants based on examined subgroups

The subgroup-based prevalence of *Blastocystis* spp. in edible plants is displayed in Table 3 and Supplementary Figs. 4–9.

**Table 3:** Subgroup analysis of *Blastocystis* spp. prevalence in edible plants according to publication year, continent, WHO region, country, plant type, sample size, and diagnostic method.

Subgroup variable	Prevalence % (95 % CI)	Heterogeneity (Q)	df (Q)	I^2^ (%)	P-value
**Publication year**					
<2015	11.2 (3.1–33.3)	53.3	2	96.2	*P* < 0.05
2015–2019	2 (0.7–6.2)	28	3	89.3	P < 0.05
2020–2024	11.9 (8–17.5)	551	19	96.5	P < 0.05

**Continent**					
Africa	8.5 (2.8–23.2)	99.4	3	97	P < 0.05
Asia	8 (4.6–13.7)	212.9	12	94.3	P < 0.05
Europe	3 (0.6–14.2)	34.1	3	91.2	P < 0.05
North America	9.3 (6.8–12.5)	0	0	0	*P* > 0.05
South America	26.5 (19.9–34.4)	52.5	4	92.4	P < 0.05

**WHO region**					
AMR	22.5 (15.5–31.6)	113.3	5	95.6	P < 0.05
EMR	6.5 (4.1–10.1)	203.7	14	93.1	P < 0.05
EUR	3 (0.6–14.2)	34.1	3	91.2	P < 0.05
SEAR	34.7 (27.1–43.2)	1.1	1	12.8	P > 0.05

**Country**					
Argentina	24.1 (19.3–29.7)	0	0	0	P > 0.05
Bangladesh	33 (26.8–39.8)	0	0	0	P > 0.05
Brazil	25.7 (14.4–41.5)	17.4	1	94.2	P < 0.05
Ecuador	33.5 (31.1–36)	0	0	0	P > 0.05
Egypt	8.5 (2.8–23.2)	99.4	3	96.9	P < 0.05
Iran	5.1 (2.6–9.7)	32.2	5	84.5	P < 0.05
Italy	0.5 (0.1–1.4)	0	0	0	P > 0.05
Lebanon	8.7 (6–12.4)	0	0	0	P > 0.05
Mexico	9.3 (6.8–12.5)	0	0	0	P > 0.05
Saudi Arabia	5.6 (1.2–22.8)	67.8	2	97	P < 0.05
Spain	1.8 (0.5–7)	0	0	0	P > 0.05
Syria	10.2 (6–16.7)	0	0	0	P > 0.05
Thailand	45 (25.3–66.4)	0	0	0	P > 0.05
Turkey	9.4 (3.1–25.1)	4	1	75.3	P < 0.05
Venezuela	22.2 (12.4–36.6)	0	0	0	P > 0.05

**Sample size**					
<200	11.6 (7–18.7)	80.4	10	87.5	P < 0.05
200–400	10.2 (5.8–17.3)	222.6	9	95.9	P < 0.05
>400	5.7 (2.1–14.2)	424.6	5	98.8	P < 0.05

**Diagnostic method**					
Mic	10 (6.8–14.4)	690.4	21	96.9	P < 0.05
Mol	6.7 (1.7–22.9)	58.2	4	93.1	P < 0.05

**Plant type**					
Fruits	12.5 (5.4–26.6)	109.8	5	95.4	P < 0.05
LGVs ^a^	9.3 (6.1–13.9)	345.8	17	95.1	P > 0.05
NLGVs ^b^	5.6 (2.5–12.1)	82	9	89	P < 0.05
Uncategorized plants	10.2 (4.5–21.5)	118.6	6	94.9	P > 0.05

^a^ LGVs: Leafy green vegetables, ^b^ NLGVs: Non-leafy green vegetables.

### 3.6 Sensitivity analysis

Based on the sensitivity analysis, excluding particular datasets on *Blastocystis* spp. in edible plants did not notably alter the overall frequency (Supplementary Fig. 10). Moreover, post-study removal, the pooled prevalence of *Blastocystis* spp. in edible plants was approximated at 8.8–10.3 %.

### 3.7 Publication bias

**Fig. 1:**
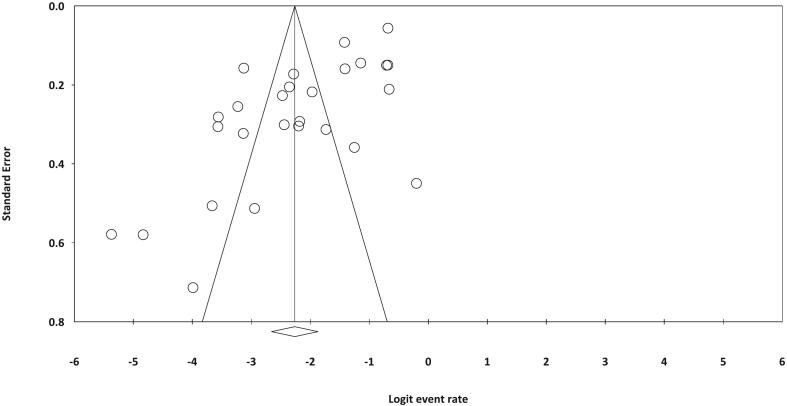
Funnel plot illustrating publication bias in the current systematic review and meta-analysis.

A significant publication bias was detected in the current systematic review and meta-analysis (Egger's regression: intercept = − 6.535, 95 % lower limit = − 9.232, 95 % upper limit = − 3.839, t-value = 4.992, *P* < 0.001) (Fig. 1).

## 4 Discussion

Foods like vegetables and fruits play a key role in transmitting pathogenic microorganisms, including parasites, to humans due to their susceptibility to contamination during production, handling, and distribution. Vegetables and fruits, for instance, can harbour parasites from the soil, water, or animal waste. Parasitic infections like giardiasis, cryptosporidiosis, toxoplasmosis, toxocariasis, and blastocystosis are some of the illnesses that can spread through tainted foods (Koutsoumanis et al., 2018; Hassanain et al., 2013; Slifko et al., 2000). Moreover, water samples can harbour nine *Blastocystis* spp. STs (ST1-ST4, ST6, ST8, ST10, ST21, and ST24), with seven (ST1-ST4, ST6, ST8, and ST10) capable of infecting humans. To address the risk posed by these parasites, it is crucial to implement preventive measures, enhance water source cleanliness, and promote public health awareness (Javanmard et al., 2019; Mahdavi et al., 2024).

The current study is the first global systematic review and meta-analysis on the prevalence and STs distribution of *Blastocystis* spp. in edible plants. Edible plants analysed for contamination with this protistan parasite encompass fruits, leafy green vegetables, and non-leafy green vegetables on a global scale.

The significance of studying *Blastocystis* spp. in food items lies in its potential pathogenicity and the distribution of its zoonotic STs. Several mechanisms have been proposed for *Blastocystis* spp. pathogenicity, including the ability of parasite to adhere to and invade the intestinal lining, release toxins, disrupt the gut microbiota, and modulate the immune response of the host. Overall, the pathogenicity of *Blastocystis* spp. is not yet well understood and is still a subject of ongoing research and debate within the scientific community (Ajjampur and Tan, 2016; Wawrzyniak et al., 2013). Some studies have proposed a connection between *Blastocystis* spp. infection and gastrointestinal symptoms like diarrhea, abdominal pain, and bloating, while others have not identified a clear association (El Safadi et al., 2016; Tan et al., 2010).

The present study found that 9.4 % (95 % CI: 6.6–13.4 %) of global edible plants contained *Blastocystis* spp. The sensitivity analysis found no outliers among the included studies, and removing individual studies did not significantly alter the final prevalence of *Blastocystis* spp. in edible plants. Fruits showed the highest rate of *Blastocystis* spp. contamination at 12.5 % (95 % CI: 5.4–26.6 %), surpassing uncategorized plants at 10.2 % (95 % CI: 4.5–21.5 %), leafy green vegetables at 9.3 % (95 % CI: 6.1–13.9 %), and non-leafy green vegetables at 5.6 % (95 % CI: 2.5–12.1 %). Previous reviews and meta-analyses on food or edible plants have not delved into individual gastrointestinal parasites in detail, but have instead focused on the overall prevalence of protozoan or helminth infections. Based on this, 20 % of vegetables and 31 % of fruits were infected with protozoan and helminthic infections, respectively (Badri et al., 2022; Eslahi et al., 2022). These results indicate a higher prevalence of parasitic infections in vegetables than in fruits. Conversely, both vegetables and fruits exhibit a higher incidence of helminth infections than protozoan infections. However, the prevalence rates in different food groups can vary depending on geographic location, irrigation and maintenance practices, environmental cleanliness, sample size, and the sensitivity of diagnostic methods.

A key issue in accurately estimating the prevalence of *Blastocystis* spp. is the detection methodology (Nemati et al., 2021). In the past decade, molecular analyses of *Blastocystis* spp. have gained popularity, with various research groups globally investigating the significance of its genetic diversity. Two primary analysis methods have been used: *SSU-rDNA* sequencing and polymerase chain reaction (PCR) amplification of sequence-tagged sites (STS). *SSU-rDNA* sequencing provides quantitative data and can detect new molecular STs as they emerge. In contrast, STS-PCR excels in identifying mixed infections by separately detecting each major ST, although it is limited to only seven known STs (Clark et al., 2013; Yoshikawa et al., 2004). Currently, various regions of the *SSU-rDNA* gene are commonly used for PCR-based identification of *Blastocystis* spp. For example, primers targeting the Santin region (Santín et al., 2011) demonstrate superior specificity and sensitivity compared to those for the Barcode and Stensvold regions (Stensvold, 2013). Additionally, Barcode region primers can inadvertently amplify *SSU-rDNA* genes from other eukaryotes, particularly fungi, in samples lacking *Blastocystis* spp., especially when directly screening fecal DNA. Barcoding is only effective for molecular characterization of known positive samples, not for screening (Stensvold and Clark, 2016a, Stensvold and Clark, 2016b).

In the current study, the presence of important zoonotic *Blastocystis* spp. STs, like ST1 and ST3, in plant-based foods suggests potential contamination from water and soil exposed to human and animal waste throughout the growth and harvesting process. This contamination can lead to parasitic infections in humans, ranging from mild to severe. Hence, maintaining sanitation, using clean water, and properly washing plant foods can greatly lower the risk of *Blastocystis* spp. infections.

A previous large-scale study demonstrated a strong association between *Blastocystis* spp. prevalence and favourable cardiometabolic outcomes (Piperni et al., 2024). Specifically, individuals exhibiting higher *Blastocystis* spp. carriage had lower obesity rates and improved gut microbial diversity, suggesting that *Blastocystis* spp. may be a marker of gut health. The study also found that dietary improvements led to an increase in *Blastocystis* spp. prevalence and abundance, reinforcing its potential link to healthier dietary patterns. A plant-based diet, which is rich in fiber and bioactive compounds, is known to promote gut microbial diversity, and this may partly explain the higher *Blastocystis* spp. presence observed in individuals with improved dietary habits. While *Blastocystis* spp. has been linked to beneficial gut microbiome profiles in humans, our systematic review and meta-analysis raise public health concerns regarding its presence in edible plants. The detection of *Blastocystis* spp. in 9.4 % of global edible plants, particularly in fruits and leafy green vegetables, highlights the potential for foodborne transmission. The presence of zoonotic STs in edible plants further underscores the risk of cross-species transmission, which may have implications for human health, particularly in immunocompromised individuals. This contrast suggests that while *Blastocystis* spp. might be a marker of a healthy gut in some individuals, its presence in food sources raises concerns about contamination, potential pathogenicity, and the need for improved food safety measures.

The dual role of *Blastocystis* spp. as both a potential health marker and a pathogen suggests its effects are context-dependent. Diet and immune status are factors influencing whether *Blastocystis* spp. acts as a commensal or pathogen (Kumarasamy et al., 2018; Lepczyńska et al., 2017). A high-fiber, plant-based diet fosters a gut environment where *Blastocystis* spp. can coexist beneficially with other microbes, while a poor diet, high in fat and low in fiber, can lead to dysbiosis. Individuals with strong immune systems may tolerate or benefit from *Blastocystis* spp., whereas those with weakened immunity might suffer gastrointestinal disturbances. Additionally, different *Blastocystis* spp. STs can have varying pathogenic potentials; for instance, ST1 and ST3 are common in both humans and animals, and their presence in edible plants raises concerns about transmission and pathogenicity in at-risk populations.

Due to variations in study diversity among different groups, caution is advised when interpreting results from subgroup analysis. Notably, there was no direct link between the year of publication and the pooled prevalence of *Blastocystis* spp. However, the highest weighted prevalence rate was seen in the years 2020–2024 [20 datasets, 11.9 % (95 % CI: 8–17.5 %)]. South America [five datasets, 26.5 % (95 % CI: 19.9–34.4 %)] and the SEAR WHO region [two datasets, 34.7 % (95 % CI: 27.1–43.2 %)] had the highest pooled prevalence of *Blastocystis* spp. Moreover, Thailand [one dataset, 45 % (95 % CI: 25.3–66.4 %)], Ecuador [one dataset, 33.5 % (95 % CI: 31.1–36 %)], and Bangladesh [one dataset, 33 % (95 % CI: 26.8–39.8 %)] reported the highest prevalence rates. An inverse association between sample size increase and reported prevalence decrease was observed, underscoring the significance of larger sample sizes for more accurate prevalence estimates. Additionally, the use of microscopic diagnostic methods and consumption of fruits showed the highest *Blastocystis* spp. prevalence rates of 10 % and 12.5 % respectively. Despite the valuable epidemiological information mentioned, the lack of studies from various regions worldwide, the reliance on one study/dataset in most subgroups, and the small number of molecular studies are limitations that could impact the results. Hence, caution is advised when interpreting the findings from this study.

## 5 Conclusions

Our findings underscore the need for a nuanced perspective on *Blastocystis* spp. While it may serve as a gut health indicator in individuals consuming plant-based diets, its presence in edible plants necessitates stringent food safety measures to prevent potential foodborne transmission. Future research should focus on determining the specific mechanisms by which *Blastocystis* spp. interacts with the gut microbiome to confer health benefits, investigating subtype-specific differences in pathogenicity and their implications for human health, and assessing the effectiveness of food safety interventions in reducing *Blastocystis* spp. contamination in the food supply chain.

## Availability of data and materials

The datasets supporting the conclusions of this article are included in the article and its additional files.

## CRediT authorship contribution statement

**Abdollah Didban:** Writing – original draft, Methodology, Investigation. **Farajolah Maleki:** Writing – original draft, Writing – review & editing. **Laya Shamsi:** Investigation, Methodology. **Ali Asghari:** Writing – review & editing, Writing – original draft, Supervision, Software, Methodology, Investigation. **Behzad Bijani:** Methodology, Investigation. **Amin Karampour:** Methodology, Investigation.

## Declaration of competing interest

The authors declare no potential conflicts of interest with respect to the research, authorship, and/or publication of this article.

## Appendix A Supplementary data

Supplementary material 1 Image 1

Supplementary material 2 Image 2

Supplementary material 3 Image 3

Supplementary material 4 Image 4

Supplementary material 5 Image 5

Supplementary material 6 Image 6

Supplementary material 7 Image 7

Supplementary material 8 Image 8

Supplementary material 9 Image 9

Supplementary material 10 Image 10

Supplementary material 11 Image 11
