# Supplementary material for: Edible plants as significant sources of Blastocystis spp. infections: A systematic review and meta-analysis
Source: Food Waterborne Parasitol. 2025 Mar 2;38:e00254. doi: 10.1016/j.fawpar.2025.e00254 (PMC11925565; doi:10.1016/j.fawpar.2025.e00254)
Supplement: Supplementary file 1 — Supplementary material 1 [file mmc1.docx]

**Identification**

**Screening**

**Eligibility**

**Included**

**Studies identified through international database search**

**(n= 7,925)**

**Studies remained after removal of the duplicates**

**(n= 4,682)**

**Studies remained for qualitative and quantitative analyses (n=27)**

**Full-text papers assessed for eligibility (n=31)**

**Studies excluded after tittle and abstract screening (n= 4,650)**

**Papers excluded for various reasons (n=4)**

**Supplementary Fig. 1.** Flowchart of the included eligible studies in the present systematic review.
