# Supplementary material for: Edible plants as significant sources of Blastocystis spp. infections: A systematic review and meta-analysis
Source: Food Waterborne Parasitol. 2025 Mar 2;38:e00254. doi: 10.1016/j.fawpar.2025.e00254 (PMC11925565; doi:10.1016/j.fawpar.2025.e00254)
Supplement: Supplementary file 3 — Supplementary material 3 [file mmc3.docx]

**Supplementary Fig. 3.** Weighted prevalence of *Blastocystis* spp. based on plant types.
