# Supplementary material for: Edible plants as significant sources of Blastocystis spp. infections: A systematic review and meta-analysis
Source: Food Waterborne Parasitol. 2025 Mar 2;38:e00254. doi: 10.1016/j.fawpar.2025.e00254 (PMC11925565; doi:10.1016/j.fawpar.2025.e00254)
Supplement: Supplementary file 8 — Supplementary material 8 [file mmc8.docx]

**Supplementary Fig. 8**. The global prevalence of *Blastocystis* spp. in edible plants based on sample size.
