# Supplementary material for: Edible plants as significant sources of Blastocystis spp. infections: A systematic review and meta-analysis
Source: Food Waterborne Parasitol. 2025 Mar 2;38:e00254. doi: 10.1016/j.fawpar.2025.e00254 (PMC11925565; doi:10.1016/j.fawpar.2025.e00254)
Supplement: Supplementary file 11 — Supplementary material 11 [file mmc11.docx]

**Supplementary Table 1**

**JBI critical appraisal checklist applied for included studies**

| Author Name/Year | Sample was representative? | Participants appropriately recruited? | Sample size was adequate? | Study subjects and the setting described? | Data analysis conducted | Objective, standard criteria, reliably used? | Appropriate statistical analysis used | Confounding factors/ subgroups/ differences identified and accounted? | Subpopulations identified using objective criteria | Overall quality |
| --- | --- | --- | --- | --- | --- | --- | --- | --- | --- | --- |
| Soares, 2006 | Yes | Yes | Yes | Yes | Yes | Yes | No | No | No | 6/9 |
| Al-Megrin, 2010 | Yes | No | Yes | Yes | No | Yes | No | No | No | 4/9 |
| Cazorla-Perfetti, 2013 | Yes | No | Yes | No | Yes | No | Yes | No | No | 4/9 |
| Rahimi Esboei, 2017 | Yes | Yes | Yes | Yes | Yes | No | Yes | Yes | No | 7/9 |
| Caradonna, 2017 | Yes | Yes | Yes | Yes | Yes | No | Yes | No | No | 6/9 |
| Etewa, 2017 | Yes | Yes | Yes | Yes | Yes | No | Yes | Yes | Yes | 8/9 |
| Taherimoghaddam, 2018 | Yes | Yes | Yes | Yes | Yes | Yes | Yes | Yes | No | 8/9 |
| Isazadeh, 2020 | Yes | Yes | Yes | Yes | Yes | No | Yes | No | Yes | 7/9 |
| Rodrigues, 2020 | Yes | Yes | Yes | Yes | Yes | No | Yes | Yes | Yes | 8/9 |
| Heidari, 2020 | Yes | No | Yes | Yes | No | Yes | Yes | No | Yes | 6/9 |
| Al Nahhas, 2020 | Yes | Yes | Yes | Yes | Yes | No | Yes | Yes | Yes | 8/9 |
| Aydoğdu, 2021 | Yes | Yes | Yes | Yes | Yes | Yes | Yes | Yes | No | 8/9 |
| Rahimi, 2021 | Yes | Yes | Yes | Yes | Yes | No | Yes | No | Yes | 7/9 |
| Abdel-Hakeem, 2021 | Yes | Yes | Yes | Yes | Yes | No | Yes | Yes | Yes | 8/9 |
| Morales-Figueroa, 2021 | Yes | No | Yes | Yes | No | Yes | Yes | No | Yes | 6/9 |
| Hussein, 2021 | Yes | Yes | Yes | Yes | Yes | Yes | No | No | No | 6/9 |
| Hoseinifard, 2022 | Yes | No | Yes | Yes | No | Yes | No | No | No | 4/9 |
| Abd-Rabou, 2022 | Yes | No | Yes | No | Yes | No | Yes | No | No | 4/9 |
| Jinatham, 2022 | Yes | Yes | No | Yes | No | Yes | No | Yes | No | 5/9 |
| El Safadi, 2023 | Yes | Yes | Yes | Yes | Yes | No | Yes | Yes | No | 7/9 |
| Falcone, 2023 | Yes | Yes | Yes | Yes | Yes | No | Yes | No | No | 6/9 |
| Moreno-Mesonero, 2023 | Yes | Yes | Yes | Yes | Yes | No | Yes | Yes | Yes | 8/9 |
| Bilgiç, 2023 | Yes | Yes | Yes | Yes | Yes | Yes | Yes | Yes | No | 8/9 |
| Barua, 2023 | Yes | Yes | Yes | Yes | Yes | No | Yes | Yes | Yes | 8/9 |
| Altwaim, 2023 | Yes | Yes | Yes | Yes | Yes | Yes | Yes | Yes | No | 8/9 |
| El-Sayed, 2023 | Yes | Yes | Yes | Yes | Yes | No | Yes | No | Yes | 7/9 |
| González-Ramírez, 2024 | Yes | Yes | Yes | Yes | Yes | No | Yes | Yes | Yes | 8/9 |
